# Biological Evaluation of Polyherbal Ayurvedic Cardiotonic Preparation “*Mahamrutyunjaya rasa*”

**DOI:** 10.1155/2011/801940

**Published:** 2010-09-02

**Authors:** Pallavi D. Rai, Sadhana J. Rajput

**Affiliations:** Quality Assurance Laboratory, Centre of Relevance and Excellence in Novel Drug Delivery System, Pharmacy Department, G. H. Patel Building, Donor's Plaza, The Maharaja Sayajirao University of Baroda, Fatehgunj, Vadodara, Gujarat 390 002, India

## Abstract

*Mahamrutyunjaya rasa* (MHR), an Ayurvedic formulation, used as cardiotonic, contains potentially toxic compounds like aconitine, which are detoxified during preparation using traditional methods. Comparative toxicological evaluation of laboratory prepared formulation (F1) and two marketed formulations (F2 and F3) were performed based on their effects on viability of H9c2 cells and after single oral dose administration in mice. Cardioprotective effect of formulations at 25 and 50 mg/kg doses were studied in isoproterenol- (ISO-) induced myocardial infarcted rats. F1 and F2 did not affect the cell viability, while F3 decreased the cell viability in concentration and time-dependent manner. Rats administered with ISO showed significant increase in the serum levels of glutamate oxaloacetate transaminase, alkaline phosphotase, creatinine kinase isoenzymes, lactate dehydrogenase, and uric acid, while F1 and F2 treatment showed significant reduction in the same. F3 showed further increase in the serum levels of enzymes and uric acid in ISO-challenged rats. High pressure liquid chromatographic analysis of formulations showed higher concentration of aconitine in F3. Study shows that F1 and F2 possess cardioprotective property with higher safety, while formulation F3 cannot be used as cardioprotective due to its cytotoxic effects. Thus, proper quality assessment methods are required during preparation of traditional formulations.

## 1. Introduction

Today, herbal remedies are back into prominence because the efficacy of conventional medicines such as antibiotics, which once had near universal effectiveness against serious infections, is on the wane. In *Ayurveda *(traditional Indian medicine) about 2,000 plant species are considered to have medicinal value, while the Chinese Pharmacopoeia lists over 5,700 traditional medicines, most of which are of plant origin [1]. 

Although modern drugs are effective in the symptomatic control of cardiovascular disease, their use is often associated with a number of undesirable effects [2]. Various ayurvedic formulations have been found to be clinically useful remedy in a number of disorders with advantages like better acceptance by the patient and less cost [3, 4]. However, the claimed pharmacological activities of many of these formulations have not been proven nor refuted by controlled studies. Present studies on ayurvedic cardiotonic formulations are of important considerations, since cardiovascular remedies are usually taken for long periods of time. MHR is a kind of compound herbo-mineral formulation often used to treat cardiac disorders [5]. The medicine comprises four kinds of herbs: *Aconitum ferox, Solanum indicum, Piper nigrum* and *Piper longum.* Ayurvedic literature [6] records the formula of MHR tablet as 1 part each of powdered processed *Aconitum ferox, Solanum indicum, Piper nigrum,* and *Piper longum,* sieved and then mixed with 1 part each of purified sulphur and purified sodium metaborate. To this mixture 2 parts of purified cinnabar (HgS) were added and mixed uniformly. In this complex formulation, the mixtures of plant constituents act synergistically to increase the activity and also at times counteract the toxic effect of compounds [7]. Aconite root contains various toxic diester-diterpene alkaloids like *Aconitine*, which is known for its cardiotonic [8], anti-inflammatory, and analgesic effects [9]. The LD_50_ of *Aconitine* in mice is 1.8 mg/kg. The purification of Aconitum roots is done by soaking the roots in cow urine for 48 h followed by washing them with cow milk. The roots have to be then washed with water and dried [10]. Solanum roots also contain toxic glycoalkaloids **α*-Solanine* with LD_50_ of 0.68 mmol/kg in mice [11]. The heavy metal content of this formulation can also present serious risk to human health [12]. *Shodhana* (purification), a traditional ayurvedic treatment was used to decrease the toxicity and increase the bioactivity of the active ingredients in the preparation of MHR [13]. If this purification is not performed as per the standard text, undesirable effects of formulation may be observed after administration in humans [14]. The toxicological and pharmacological properties of MHR have not been subjected to any scientifically controlled investigations so far. Thus, it becomes important for the physicians and producers to be aware of the safety and efficacy of MHR. The present investigations have therefore been designed to explore the toxicological and pharmacological effects of MHR to substantiate the claims made in Ayurveda as well as by traditional healers and provide a platform to prove the safety of such formulations.

In this study we investigated the cardioprotective action of various MHR formulations in experimentally induced cardiac damage. We used Isoproterenol (ISO) (1-(3′,4′-dihydroxyphenyl)-2-isopropylaminoethanol hydrochloride), a nonselective *β*-adrenergic agonist, which causes infarct-like necrosis of the heart muscle with subsequent leakage of cardiac enzymes in blood leading to cardiac dysfunction in rats [15–17]. Cardioprotective action of MHR was studied in ISO-challenged rats. We further studied cytotoxicity of MHR formulation using rat embryonic cardiac cell line (H9c2). Chromatographic analysis of selected MHR formulations was performed in order to validate the purification process of one of the ingredients used during its preparation.

## 2. Experimental

The present study was performed on biological and toxicological aspects of MHR. Three formulations (F1, F2, and F3) were selected. F1 was prepared in laboratory as reported in the traditional text; F2 was procured from Baidyanath, (Nagpur, India), while formulation F3 was purchased from Pune Rasashala, (Pune, India). 

### 2.1. Chemicals and Reagents

Dulbecco's Modified Eagle's Medium (DMEM) and fetal calf serum (FCS) were obtained from GIBCO, Invitrogen Corporation, Carlsbad, California. 3-(4, 5-dimethylthiazol-2-yl)-2, 5-diphenyltetrazolium bromide (MTT), streptomycin, isoproterenol, and trypsin were purchased from Sigma Chemical Co. (St. Louis, MO, USA). HPLC grade acetonitrile and methanol and analytical grade Potassium dihydrogen phosphate (KH_2_PO_4_), triethylamine, and 85% *ortho*-phosphoric acid (H_3_PO_4_), were purchased from (Qualigens, Mumbai). Triple distilled water was used throughout the study. Serum GOT and ALK-P and uric acid detection kits were purchased from (Sigma Diagnostic Kit, Sigma-Aldrich Corp., St. Louis, MO), while LDH detection kit from (Teco Diagnostics, Anaheim, CA, USA). All other chemicals used were of the highest grade available.

### 2.2. Sample Preparation

The formulations in tablet form (F2 and F3) were purchased from the local market. The tablets were powdered and suspended in water using 1% tween 80 for the *in vivo* studies. Alkaloid fractions of the formulations (F1, F2, and F3) were prepared as per the reported method [18] and fractions were dried under high vacuum for several hours to remove any traces of solvents used during their processing. The dried fractions were dissolved in dimethyl sulfoxide (DMSO) in aseptic conditions and filtered through 0.25 *μ*m syringe filter and then used for *in vitro* studies. The final concentration of DMSO was not more than 0.2% during the experiments.

### 2.3. Animals

Female Balb/c mice weighing between 20–25 g and male albino rats of Wistar strain weighing 250 to 280 g (Cadila Pharmaceuticals, Ahmedabad, India) were housed for at least 4 days before being used in a room with controlled temperature (23 ± 1°C), humidity (50 ± 10%) and light (6 : 00 A.M. to 6 : 00 P.M.). Food and water were made available continuously. Animal care and use for experimental procedures were approved by the Institutional animal care and use committee. All anesthetic and procedures were in compliance with the guidelines established by the Animal Care Committee (Pharmacy department/M. S. University, Baroda/404/01/1/CPCSEA).

### 2.4. Cell Culture and Treatment

The H9c2 cell line derived from embryonic rat heart tissue was purchased from America Tissue Type Collection (Manassas, VA; catalog # CRL-1446). Cells were cultured in DMEM supplemented with 10% FCS, 100 U/mL of penicillin, and 100 *μ*g/mL of streptomycin in 75 cm^2^ tissue culture flasks at 37°C in a humidified atmosphere of 5% CO_2_. Alkaloidal fractions of formulations dissolved in DMSO were suspended in sterile DMEM for *in vitro* assay.

### 2.5. Single Dose Toxicity of Formulations in Mice

Toxicity study with single oral doses of formulations was carried out as per the OECD guidelines using mice [19]. Mice were administered orally with single dose of 2000, 1550, 550, 175, 55 mg/kg of formulations and observed for any signs of toxicity and mortality. Histopathological examination of the visceral organs like heart, kidney, and liver was done in order to observe the toxic effects of formulations [20].

### 2.6. Cell Viability Study of Formulations in Rat Embryonic Cardiac Cells

The viability of H9c2 cells after treatment with alkaloidal fractions of formulations F1, F2, and F3 was assayed by the reduction of MTT to formazan as described previously [21]. The cells (5 × 10^3^) were cultured in 96—well microtiter plates, and left overnight in an incubator at 37°C with 5% CO_2_ before being exposed to different concentrations of formulations. Cells were treated with different concentrations (2, 5, 10, 20, 50, 100, and 200 *μ*g/mL) of F1, F2, and F3 for 12, 24, and 48 h and their viability was determined using 10 *μ*L of 5 mM MTT with additional incubation for 4 h. Thereafter, the medium was removed, the formazan crystals were dissolved in 100 *μ*L of DMSO and the absorbance was measured at 570 nm using microplate reader (Molecular Devices, Spectra MAX 250). The data of the survival curves were expressed as the percentage of untreated controls [22].

### 2.7. Protective Effect of Formulations against ISO-Induced Myocardial Infarction (MI) in Rats

Cardioprotective effect of formulations was studied in ISO-induced myocardial infarcted rats. Rats were administered with 25 and 50 mg/kg doses of formulations orally for 15 days and at the end of treatment MI was induced by injecting 25 mg/kg s.c. dose of ISO twice at the interval of 24 h. The protective effect of formulations was evaluated by comparing the results with vehicle-treated rats injected with ISO. The experimental design for the study has been shown in Table 1. At the end of experiment, rats were sacrificed under ether anesthesia, blood was collected through carotid artery, and serum was separated. Serum levels of cardiac marker enzymes like lactate dehydrogenase (LDH), creatine kinase isoenzymes (CK-MB), glutamate-oxaloacetate transaminase (GOT), and alkaline phosphatase (ALK-P) along with uric acid was determined in order to evaluate the cardiac injury. Serum ALK-P, GOT were determined using commercial kits (Sigma Diagnostic Kit, Sigma-Aldrich Corp., St. Louis, MO) [23, 24], while serum levels of uric acid were determined by spectrophotometric assay (Sigma, St. Louis, MO) [25]. CK-MB activity was determined with a colorimetric method using a CK-MB immunoinhibition kit (Euro Diagnostic System Pvt. Ltd, Chennai, India), based on the principle of immunoinhibition of one of the subunits (CK-M and CK-B) of the enzyme creatine kinase [26]. The enzyme LDH catalyzes the oxidation of lactate to pyruvate in the presence of NAD, which is subsequently reduced to NADH. The rate of NADH formation measured at 340 nm is directly proportional to the serum LDH activity, based on this principle we measured serum levels of LDH using commercially available kit (Teco Diagnostics, Anaheim, CA, USA) [27]. Heart was dissected out, washed in ice-cold saline and weighed accurately to determine heart weight/body weight ratio (HW/BW). Histopathological studies on heart were carried out in order to assess any changes in cellular architecture. The body weights of the animals were recorded throughout the experimental period.

### 2.8. Chromatographic Analysis

The High Pressure Liquid chromatography (Shimadzu, Kyoto, Japan) consisted of LC-20 AT Prominence solvent delivery module (Shimadzu), a manual injector with a 20 *μ*L fixed loop (Rheodyne), and a SPD-20A Prominence UV-visible detector (Shimadzu) was used to determine the concentration of active ingredient in the formulations. The separation was performed on a C_18_ column (particle size 5 *μ*m; 250 mm ×  4.6 mm ID; Phenomenex Torrance, USA) preceded by an ODS guard column (10 *μ*m, 10 mm × 5 mm ID) at an ambient temperature. Chromatographic data were recorded and processed using a Chromatographic Station CFR Version 2.4.0.193 (Spinchrom Pvt. Ltd., Chennai, India).

### 2.9. Preparation of Standard Solutions

Standard stock solutions of *aconitine* (1000 *μ*g/mL) were prepared by dissolving 10 mg of pure *aconitine* in 10 mL methanol. Further dilutions of standards were made by using aliquots of the stock and making volume with methanol in order to obtain the concentration of 10–100 *μ*g/mL.

### 2.10. Preparation of Sample Solutions

Twenty tablets of all the three formulations were powdered and about 1 gm each of the three formulations was accurately weighed and extracted in 25 mL of 0.1 N HCl by sonication for 10 minutes at room temperature. The solutions were further fractionated with 10 × 3 mL ethyl acetate to remove the nonalkaloid components. The acidic aqueous solutions were basified using ammonia to pH 11 and further extracted with 10 × 3 mL chloroform. Chloroform was evaporated under reduced pressure and residue obtained was dissolved in methanol by sonication and further dilutions were made in acetonitrile [28].

### 2.11. Analytical Conditions

Analysis of *aconitine* in formulations was isocratic at 1.0 mL/min flow rate with ammonium bicarbonate buffer (15 mM, pH 7.5 was adjusted using ammonia): acetonitrile (40 : 60 v/v) as mobile phase. The analysis was performed at 223 nm [29].

### 2.12. Statistical Analysis of Data

Data are presented as Mean ± S.E.M. The significance of differences was estimated by one-way analysis of variance followed by application of the Dunnett's Multiple Comparisons and Tukey-Kramer Multiple Comparisons test. A *P* value of less than.05 was considered to be significant. The statistical analysis was processed with GraphPad Prism software Version 5.00 (GraphPad, San Diego, CA, USA).

## 3. Results

### 3.1. Toxicity of Formulations in Mice at Single Dose

**Figure 1:**
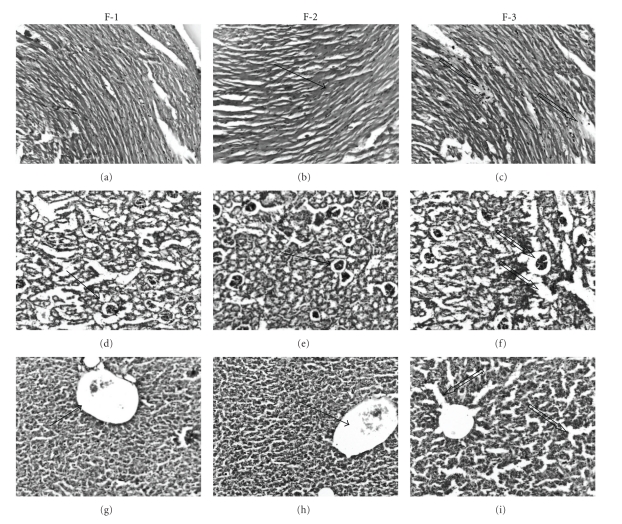
Microscopic images of mice heart, kidney, and liver illustrating effect of single oral dose administration of formulation F1, F2, and F3 (Staining: Hematoxylin and Eosin). Microscopic images of mice heart from (a) F1, (b) F2, (c) F3 groups; images of mice kidney from (d) F1, (e) F2, and (f) F3 images of mice liver from (g) F1, (h) F2, and (i) F3 groups. Solid arrow indicates the normal; and unfilled arrow indicates the affected/infracted/injured area of kidney/heart//liver.

Mortality was observed in mice administered with single oral dose (2000 and 1550 mg/kg) of F1, F2, and F3, while with 550 mg/kg dose no mortality was seen. Histopathological examination of the liver, heart, and kidney from the mice treated with 550 mg/kg dose of F1- and F2-showed normal cellular architect indicating lack of any toxicity. Liver histology of F-1 and F2-treated mice showed normal cellular architecture with distinct hepatic cells, sinusoidal spaces, and prominent central vein (Figures 1(g), 1(h)). Heart and Kidney sections were free of pathological indications in F1- and F2-treated groups of mice (Figures 1(a), 1(b), 1(d), 1(e)). Mice treated with 550 mg/kg of F3 showed disturbed liver architect. The hepatic cells appeared as cloudy swelling with branching of central portal vein. Several necrotic areas were observed in liver from F3-treated mice (Figure 1(i)). Histological analysis of Kidney from F3-treated mice exhibited disturbed cellular lining, with elongated tubules. Most of renal tubules were dilated and the epithelial cells tended to be vacuolated with a foamy appearance (Figure 1(f)). Heart sections from F3-treated mice showed infarcted zone with occurrence of oedema and inflammatory cells. Loss of contractile bands with occurrence of necrotic areas and displaced nuclei was seen in F3 treated mice. However, no mortality was observed (Figure 1(c)). Thus, formulation F3 was found to be toxic. Further toxicological studies were carried out using rat embryonic cardiac cell line (H9c2).

### 3.2. Study on Effect of Formulation on Viability of H9c2 Cells

**Figure 2:**
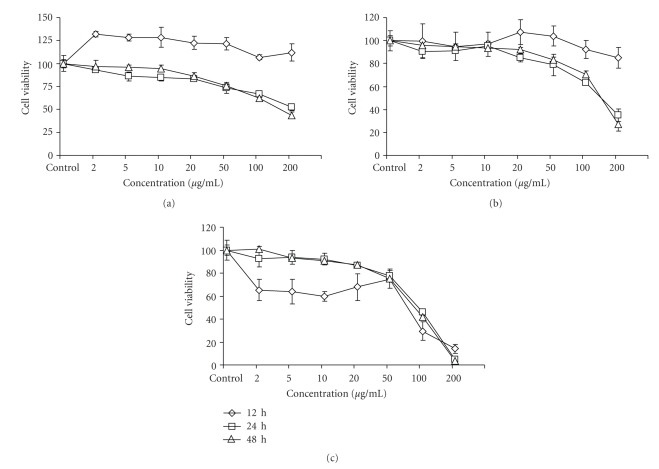
Effect of formulations F1 (a), F2 (b), and F3 (c) on viability of H9c2 cells after 12, 24, and 48 h of treatment. Values are expressed as mean ± SD and are average of three determinations.

Effect of formulations F1, F2, and F3 on viability of H9c2 cells were studied in comparison to the untreated (control) cells. Cells incubated with F1 (2 to 200 *μ*g/mL) for 12 h showed increased cell viability. Significant decrease in cell viability was observed after 24 and 48 h of treatment with maximum decrease of 47.49% at 24 h and 56.34% at 48 h with 200 *μ*g/mL concentration (Figure 2(a)).

Formulation F2 showed 14.85% decrease in cell viability at 200 *μ*g/mL concentration after 12 h of treatment, while treatment for 24 and 48 h decreased cell viability by 65.08% and 72.66% with 200 *μ*g/mL concentrations, respectively, (Figure 2(b)).

Treatment with formulation F3 decreased cell viability by 34.71, 36.08, 40.42, 31.97, 25.35, 70.55, and 85.55% after 12 h of treatment; similarly 24 h treatment decreased cell viability by 7.68, 6.49, 7.94, 13.63, 22.09, 53.84, and 94.98% at 2, 5, 10, 20, 50, 100, and 200 *μ*g/mL concentrations respectively. Cell viability was decreased by 7.23, 9.23, 13.01, 24.48, 58.51, and 96.15% with 5, 10, 20, 50, 100, and 200 *μ*g/mL concentrations respectively, after 48 h of treatment (Figure 2(c)).

### 3.3. Study on Efficacy of F1, F2, and F3 on ISO-Induced MI

Formulations F1, F2, and F3 were studied for their protective effect against ISO-induced MI in rats at 25 mg/kg and 50 mg/kg doses. Group II (vehicle-treated rats injected with 25 mg/kg ISO subcutaneously) showed significant increase (*P* < .001) in serum levels of CK-MB, LDH, GOT, ALK-P and uric acid compared to Group I (vehicle-treated rats injected with normal saline 2 mL/kg). Group IV and V (administered with 25 and 50 mg/kg doses of F1), showed significant decrease (*P* < .001) in the serum levels of CK-MB, LDH, GOT, ALK-P and uric acid in a dose dependent manner compared to Group II (vehicle-treated rats injected with ISO). Group III (administered with 50 mg/kg dose of F1 alone) showed no change in the levels of these enzymes compared to Group I.

Group VII and VIII (administered with 25 and 50 mg/kg doses of F2, resp.) showed significant decrease (*P* < .001) in ISO-affected levels of CK-MB, LDH, GOT, ALK-P, and uric acid in a dose-dependent manner compared to Group II (vehicle-treated group injected with ISO), while Group VI (administered with 50 mg/kg dose of F2 alone) showed no change in CK-MB, LDH, GOT, ALK-P, and uric acid compared to Group I.

**Figure 3:**
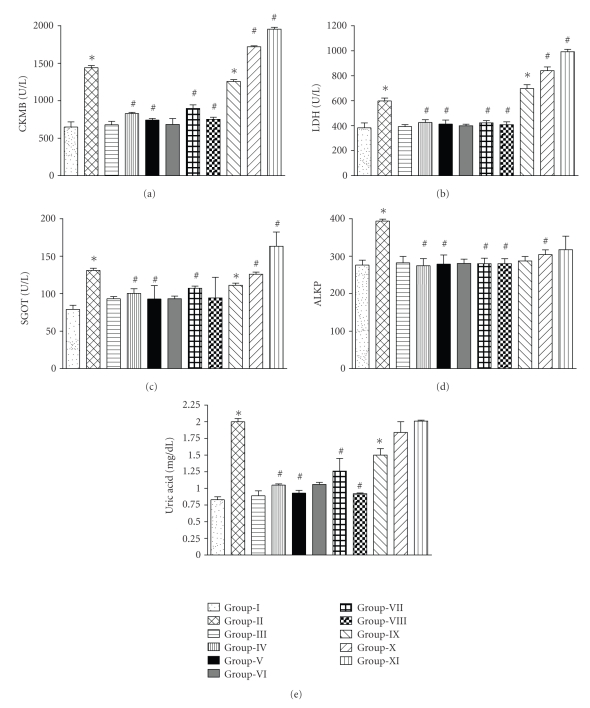
Effect of Formulations on serum levels of (a) CK-MB, (b) LDH, (c) GOT, (d) ALKP, and (e) Uric acid. Group I (1% solution of tween 80 in water followed by normal saline (2 mL/kg, s.c. twice at an interval of 24 h)); Group II (1% tween 80 followed by ISO (25 mg/kg, s.c. twice at an interval of 24 h)); Group III, VI, IX: (50 mg/kg dose of F1, F2, and F3, resp., followed by normal saline (2 mL/kg, s.c. twice at an interval of 24 h)); Group IV, VII, X: (administered orally with 25 mg/kg dose of F1, F2, and F3, respectively, followed by ISO (25 mg/kg, s.c. twice at an interval of 24 h)); Group V, VIII, XI (administered orally with 50 mg/kg dose of F1, F2, and F3, resp., followed by ISO (25 mg/kg, s.c. twice at an interval of 24 h)). #Compared with ISO treated group, *Compared with saline treated (control) group.

Group X and XI (administered with 25 and 50 mg/kg doses of F3, resp.) showed significant increase in ISO affected levels of CK-MB (*P* < .01), LDH (*P* < .001), GOT (*P* > .05), ALK-P (*P* < .001), and uric acid (*P* > .05) compared to Group II. Group IX (administered with 50 mg/kg dose of F3 alone) showed significant increase in levels of CK-MB (*P* < .001), LDH (*P* < .001), GOT (*P* < .001), ALK-P (*P* > .05), and uric acid (*P* < .01) compared to Group I. (Figure 3)

### 3.4. Heart Weight/Body Weight Ratio of the Groups

**Figure 4:**
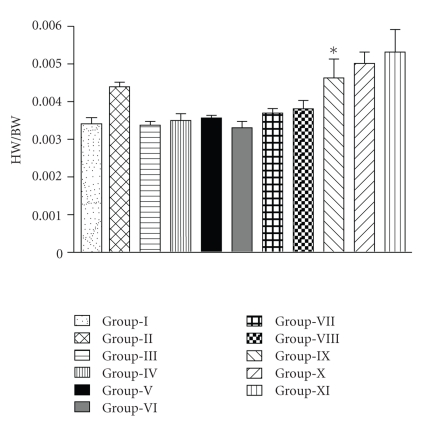
Effect of formulations F1, F2, and F3 (a) 25 mg/kg and (b) 50 mg/kg on Heart weight/body weight ratio (HW/BW). Group I (1% solution of tween 80 in water followed by normal saline (2 mL/kg, s.c. twice at an interval of 24 h)); Group II (1% tween 80 followed by ISO (25 mg/kg, s.c. twice at an interval of 24 h)); Group III, VI, IX: (50 mg/kg dose of F1, F2, and F3, resp., followed by normal saline (2 mL/kg, s.c. twice at an interval of 24 h)); Group IV, VII, X: (administered orally with 25 mg/kg dose of F1, F2, and F3, resp., followed by ISO (25 mg/kg, s.c. twice at an interval of 24 h)); Group V, VIII, XI (administered orally with 50 mg/kg dose of F1, F2, and F3, resp., followed by ISO (25 mg/kg, s.c. twice at an interval of 24 h)). *Compared with control group.

Group II showed significant increase (*P* < .01) in heart weight/body weight ratio compared to Group I. Group IV, V, VII, VIII (F1- and F2-treated groups injected with ISO) showed significant reduction in the ratio compared to Group II. Heart weight/body weight ratio was significantly increased in F3-treated group compared to Group I and Group II (Figure 4).

### 3.5. Histopathological Studies

**Figure 5:**
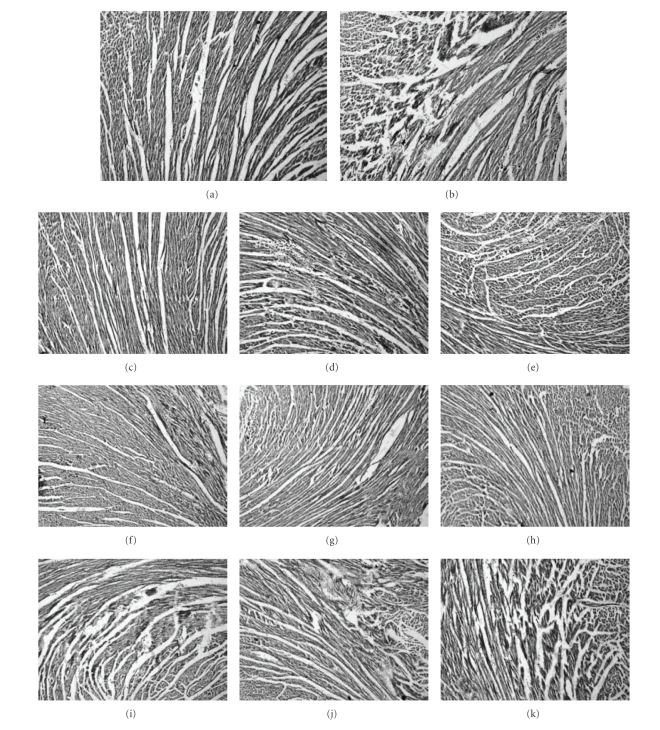
Microscopic images of rat heart illustrating the effect of formulation F1, F2, and F3 in ISO-induced MI rats (Staining: Haematoxylin and Eosin). Microscopic images of rat heart from Group I (a), Group II (b), Group III (c), Group IV (d), Group V (e), Group VI (f), Group VII (g), Group VIII (h), Group IX (i), Group X (j), Group XI (k).

Cardiac histology of rats from Group I showed normal myocardial architecture with intact muscle fibres (Figure 5(a)). Group II showed necrosis of cardiac muscles with hyalinization of fibres and cellular infiltration (Figure 5(b)). Preinfarction stage was observed in Group II as seen with occurrence of cellular inflammatory exudate, muscle atrophy, and cytoplasmic vacuolization. Cardiac muscle fibres were found to be elongated with occurrence of contractile band lesions. Cardiac histology of rats from Group III and Group VI were similar to that of the Group I (Figures 5(c), 5(f)), with minimal damage and mild swelling of muscle cells and cardiac muscle fibres, indicating lack of toxicity with formulations F1 and F2. Groups IV, V, VII, VIII administered with different doses of formulations F1 and F2 showed protection against ISO induced MI as observed from reduction in necrotic areas (Figures 5(d), 5(e), 5(g), and 5(f)). Conspicuous damage of the myocardium was observed in rats from Group X and XI (Figure 5(i)). The rats also showed acute myocardial necrosis consisted of variably sized areas of fragmented myofibers with inflammatory infiltrate (Figures 5(j), 5(k)).

Treatment with formulations F-1 and F-2 retains the normal architecture of the myocardium after ISO challenge, while F-3 failed to preserve the normal cellular architecture and proved to be cardiotoxic.

### 3.6. High-Performance Liquid Chromatography Analysis

**Figure 6:**
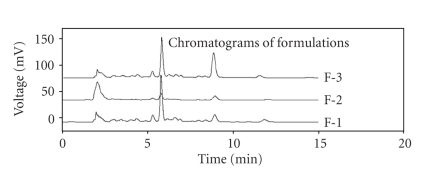
Representative chromatogram of formulations F1 (prepared in laboratory), F2 (Baidyanath), and F3 (Pune Rasashala). Retention time of Aconitine ~8.2 mins. (Aconitine content (*μ*gg^−1^) in F1-1.06, F2-1.27, and F3-5.10.).

The amount of *aconitine* was estimated in formulations F1, F2, and F3. The retention time of *aconitine* was found to be 8.2 minutes. The peak areas and peak heights were recorded. *Aconitine* content of formulation F3 was much higher compared to F1 and F2. The data (Figure 6 and Table 2) showed the correlation between the toxicity profile and concentration of the *aconitine*, indicating improper method of preparation.

## 4. Discussion

*Mahamrutyunjaya rasa* (MHR) is a cardiotonic herbo-mineral formulation containing large number of ingredients, which need to be purified and detoxified by *Shodhana* before being incorporated into the formulation [30]. These ingredients may prove toxic if not processed according to the standard text. Due to lack of investigations on the toxicity profile and evidences supporting the pharmacological activity, we performed various *in vitro* and *in vivo* studies on MHR to validate its traditional method of preparation and to provide an important standardization tools for its stringent control.

Our studies indicate higher safety margin for formulations F1 (prepared in the laboratory as per the standard text) and F2 (procured from Baidyanath) as observed from results of cell viability assay and acute toxicity study in mice. Formulation F3 showed significant toxicity in *in vitro* and *in vivo *studies. The viability of H9c2 cells decreased significantly even with lower concentrations of F3 and its acute administration in mice significantly affected the normal cardiac cellular architecture indicating its toxic nature.

In order to evaluate the cardioprotective effect of formulations F1, F2, and F3,* in vivo* studies were carried out using ISO-induced MI in rats. Myocardial necrosis induced by ISO is probably due to a primary act on the sarcolemmal membrane, followed by stimulation of adenylate cyclase, activation of Ca^2+^ and Na^+^ channels, exaggerated Ca^2+^ inflow, excess of excitation-contraction coupling mechanism, energy consumption, and cellular death [16]. Increased serum levels of CK-MB, LDH, ALK-P, GOT and uric acid are the diagnostic indicators of ISO-induced MI. An increase in the activity of these enzymes in serum is due to their leakage from myocytes as a result of necrosis induced by ISO. Increase in serum uric acid could be due to excessive degradation of purine nucleotides and proteolysis. Cardioprotective effects of formulations were assessed by analyzing the levels of serum marker enzymes like GOT, ALK-P, CK-MB, LDH, and uric acid. 

In the above study, ISO treatment in vehicle treated rats resulted in a marked elevation of CK-MB level, while F1 and F2 treatment prevented the maximum increase of CK-MB during the peak infarction in the tissues. Moreover, the activities of other cardio-specific enzyme-like LDH, GOT, and ALK-P in the serum were also found to be reduced with F1 and F2 treatment in MI rats.

 The cardioprotective action of MHR may be due to a number of components present in the formulation. *Aconitum* roots containing the diterpenoid alkaloids-like *aconitine* can be one of the ingredients recognized for the cardiac action. *Aconitine* has been reported to have a positive inotropic effect by elevating the intracellular Ca^2+^ concentration through different subcellular mechanisms which lead to an increase in Ca^2+^ transients in myocardial cells [31, 32]. The cardiotonic effect of MHR may be attributed to Ca^++^ mobilizing property of diterpene alkaloid *aconitine*. 

A significant increase in the serum marker enzymes levels in rats treated with F3 alone shows its cardiotoxic nature. The ratio of heart weight to body weight also increased significantly with F3 treatment alone depicting the increase in heart size due to inflammation. The reason for cardiotoxicity of F3 may be aggravation of intracellular Ca^2+^ occurring due to the presence of high *aconitine* content. The HPLC analysis showed that concentration of *aconitine* was within acceptable limits in formulations F1 and F2, while it was much higher in F3 making it toxic. 

**Figure 7:**
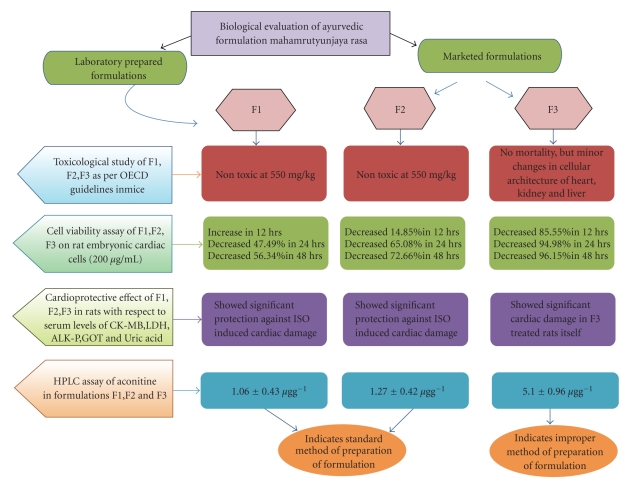
Schematic Representation of the study performed for the biological evaluation of *Mahamrutyunjaya rasa*.

As mentioned earlier, the preparation of MHR involves purification of aconite roots by treatment with cow urine in order to convert the poisonous diterpene alkaloid-like *aconitine* to other monoterpene alkaloid-like benzoylaconine which possesses the same pharmacological activity without any toxicity [10]. High *aconitine* content due to improper purification may be one of the reasons for the toxic nature of formulation F3. The presence of a number of other phyto-chemicals and heavy metals makes it further complicated to determine the actual quality of MHR formulation. The formulations F1 and F2 have proven to be safe indicating that a standard procedure has been followed for the preparation of these formulations. Thus, stringent regulatory control on the method of preparation of MHR is required to prove them safe for therapeutic use. A schematic representation of the evaluation performed on the three formulations has been shown in Figure 7. 

Since proprietary Ayurvedic medicines containing aconite roots are becoming increasingly more popular as a medicine used in the global market, methods for standardization of those medicines are in demand. The quantitation results showed that the contents of *aconitine* were significantly varied in the three MHR formulations. So, it is highly recommended that the determination of *aconitum* alkaloids in the proprietary Ayurvedic medicines must be performed as a routine measurement, so as to provide a safe application to patients in clinics, and good manufacturing practices.

To establish the potential of Ayurvedic medicine, research needs to be conducted on different disciplines of Ayurveda to meet the requirement of the society. This can be done by standardization of raw materials, methods, and measures for preparation, preservation, presentation, and administration of Ayurvedic drugs. Thus, the rationale and judicious use of modern scientific methods pertain to the development of Ayurveda.

## 5. Conclusion

It is evident from the results that MHR possess significant cardiotonic property. But, the differences in the results of toxicological and pharmacological studies prove the need of stringent regulatory control over the manufacture and quality control of this ayurvedic formulation. The alternative medicines in a number of countries have been banned owing to the toxicity due to improper processing of the components and lack of quality control standards. Thus a need arises for the development of reliable standardization tools for effective utility of these traditional medicines.

## Figures and Tables

**Table 1:** Experimental Design for the determination of protective effect of formulations against ISO-induced MI in rats.

Groups	No. of Animals	Treatment
Group I	6	1% solution of tween 80 in water for 15 days followed by normal saline (2 mL/kg, s.c. twice at an interval of 24 h)
Group II	6	1% tween 80 for 15 days followed by ISO (25 mg/kg, s.c. twice at an interval of 24 h)
Group III	6	50 mg/kg of F1 for 15 days followed by normal saline (2 mL/kg, s.c. twice at an interval of 24 h);
Group IV	6	25 mg/kg of F1 for 15 days followed by ISO (25 mg/kg, s.c. twice at an interval of 24 h)
Group V	6	50 mg/kg of F1 for 15 days followed by ISO (25 mg/kg, s.c. twice at an interval of 24 h).
Group VI	6	50 mg/kg of F2 for 15 days followed by normal saline (2 mL/kg, s.c. twice at an interval of 24 h);
Group VII	6	25 mg/kg of F2 for 15 days followed by ISO (25 mg/kg, s.c. twice at an interval of 24 h)
Group VIII	6	50 mg/kg of F2 for 15 days followed by ISO (25 mg/kg, s.c. twice at an interval of 24 h).
Group IX	6	50 mg/kg of F3 for 15 days followed by normal saline (2 mL/kg, s.c. twice at an interval of 24 h);
Group X	6	25 mg/kg of F3 for 15 days followed by ISO (25 mg/kg, s.c. twice at an interval of 24 h)
Group XI	6	50 mg/kg of F3 for 15 days followed by ISO (25 mg/kg, s.c. twice at an interval of 24 h).

**Table 2:** Contents of the *aconitine* in formulations F1, F2, and F3 (*n* = 3).

Formulation	*Aconitine* Content (*μ*gg^−1^)
F-1	1.06 ± 0.43
F-2	1.27 ± 0.42
F-3	5.10 ± 0.96
